# *In utero* exposure to cigarette chemicals induces sex-specific disruption of one-carbon metabolism and DNA methylation in the human fetal liver

**DOI:** 10.1186/s12916-014-0251-x

**Published:** 2015-01-29

**Authors:** Amanda J Drake, Peter J O’Shaughnessy, Siladitya Bhattacharya, Ana Monteiro, David Kerrigan, Sven Goetz, Andrea Raab, Stewart M Rhind, Kevin D Sinclair, Andrew A Meharg, Jörg Feldmann, Paul A Fowler

**Affiliations:** Endocrinology Unit, University/BHF Centre for Cardiovascular Science, University of Edinburgh, Queen’s Medical Research Institute, 47 Little France Crescent, Edinburgh, EH16 4TJ UK; Institute of Biodiversity, Animal Health & Comparative Medicine, College of Medical, Veterinary & Life Sciences, University of Glasgow, Glasgow, G61 1QH UK; Division of Applied Health Sciences, Aberdeen Maternity Hospital, University of Aberdeen, Foresterhill, Aberdeen, AB25 2ZD UK; TESLA (Trace Element Speciation Laboratory) and Marine Biodiscovery Laboratory, University of Aberdeen, Aberdeen, Scotland AB24 3UE UK; The James Hutton Institute, Craigiebuckler, Aberdeen, AB15 8QH UK; School of Biosciences, Sutton Bonington Campus, University of Nottingham, Loughborough, LE12 5RD UK; Institute for Global Food Security, Queen’s University Belfast, David Keir Building, Malone, Road, Belfast, BT9 5BN UK; Division of Applied Medicine, Institute of Medical Sciences, University of Aberdeen, Foresterhill, Aberdeen, AB25 2ZD UK

**Keywords:** DNA methylation, Liver, Maternal smoking, Vitamin B12

## Abstract

**Background:**

Maternal smoking is one of the most important modifiable risk factors for low birthweight, which is strongly associated with increased cardiometabolic disease risk in adulthood. Maternal smoking reduces the levels of the methyl donor vitamin B12 and is associated with altered DNA methylation at birth. Altered DNA methylation may be an important mechanism underlying increased disease susceptibility; however, the extent to which this can be induced in the developing fetus is unknown.

**Methods:**

In this retrospective study, we measured concentrations of cobalt, vitamin B12, and mRNA transcripts encoding key enzymes in the 1-carbon cycle in 55 fetal human livers obtained from 11 to 21 weeks of gestation elective terminations and matched for gestation and maternal smoking. DNA methylation was measured at critical regions known to be susceptible to the *in utero* environment. Homocysteine concentrations were analyzed in plasma from 60 fetuses.

**Results:**

In addition to identifying baseline sex differences, we found that maternal smoking was associated with sex-specific alterations of fetal liver vitamin B12, plasma homocysteine and expression of enzymes in the 1-carbon cycle in fetal liver. In the majority of the measured parameters which showed a sex difference, maternal smoking reduced the magnitude of that difference. Maternal smoking also altered DNA methylation at the imprinted gene *IGF2* and the glucocorticoid receptor (GR/*NR3C1*).

**Conclusions:**

Our unique data strengthen studies linking *in utero* exposures to altered DNA methylation by showing, for the first time, that such changes are present in fetal life and in a key metabolic target tissue, human fetal liver. Furthermore, these data propose a novel mechanism by which such changes are induced, namely through alterations in methyl donor availability and changes in 1-carbon metabolism.

**Electronic supplementary material:**

The online version of this article (doi:10.1186/s12916-014-0251-x) contains supplementary material, which is available to authorized users.

## Background

Exposure to an adverse environment *in utero* leading to a reduction in birthweight is associated with a marked increase in later susceptibility to cardiometabolic and neuroendocrine disorders [1]. Smoking in pregnancy reduces birthweight, body length, and head circumference at term [2] and is associated with increased cardiovascular disease risk in the offspring [3,4]. Thus, in addition to being one of the most important modifiable risk factors for low birthweight, maternal smoking is potentially a critical risk factor for a substantial burden of non-communicable disease in adulthood. Despite public health advice, the prevalence of smoking during pregnancy remains high: in developed countries up to 25% of pregnant women smoke and fewer than 4% stop smoking while pregnant [5].

There is growing interest in the concept that alterations in the epigenome, particularly changes in DNA methylation, may be an important mechanism linking the early life environment with later disease risk. Smoking affects DNA methylation in adults [6] and recent studies in children and adults exposed to cigarette smoke *in utero* have reported alterations in global and site-specific DNA methylation [7-10]. One potential pathway by which exposure to adverse environmental conditions, including cigarette smoke *in utero*, might impact on DNA methylation is through altered availability of methyl donors provided by the 1-carbon pathway [11]. In animal models, the availability of methyl donors and 1-carbon substrates and co-factors, such as vitamin B12, during pregnancy is associated with differences in DNA methylation in the offspring [12,13], and this may also be relevant to humans [14,15]. Maternal vitamin B12, a key co-factor in the 1-carbon cycle, is the major determinant of neonatal vitamin B12 concentrations [16] and maternal smoking reduces the concentrations of vitamin B12 in pregnant women [17] and their infants [18].

Based on initial observations of depleted concentrations of cobalt (the central metal ion in vitamin B12) in second-trimester human fetal livers, reported for the first time in this article, we hypothesized that exposure to maternal cigarette smoking would also reduce fetal vitamin B12 levels. This, in turn, may be associated with changes in 1-carbon metabolism and with DNA methylation in the fetal liver – a key metabolic target tissue. Furthermore, since exposure to maternal smoking during pregnancy has sex-specific effects on fetal growth [19,20], the expression of steroidogenic and metabolic genes in fetal liver [21,22] and on the later risk of being overweight/obese [23], we postulated that any effects would be sex-specific. We sought to test these hypotheses directly through study of the effects of prenatal smoke exposure on the fetal liver.

## Methods

### Sample collection

The collection of fetal material was approved by the NHS Grampian Research Ethics Committees (REC 04/S0802/21). Women seeking elective, medical, terminations of pregnancy were recruited with full written, informed, consent by nurses working independently at Aberdeen Pregnancy Counselling Service. There was no change in patient treatment or care associated with recruitment to the study and women were able to withdraw from the study at any point. Fetal normality was determined at ultrasound scan 2 to 9 days prior to the termination of pregnancy. Women bearing abnormal fetuses were not included in the study. Only fetuses from normally-progressing pregnancies, from women over 16 years of age with a good understanding of English, and between 11 to 21 weeks of gestation, were collected. Fetuses were transported to the laboratory within 30 minutes of delivery, weighed, crown-rump length recorded, and sexed. Blood samples were collected by cardiac puncture *ex vivo* and plasma was initially stored at −20°C and then transferred to −85°C after assay. Livers were snap-frozen in liquid nitrogen and stored at −85°C.

### Study design

For local ethical and logistic reasons it is not possible to prospectively collect specific fetuses for each study; therefore, studies were performed retrospectively by rigorous selection of already collected fetal tissues, samples, and data. To ensure accurate classification of fetuses, exposure to cigarette smoke was determined by measuring fetal plasma cotinine [24]. From a population of 228 fetuses available, two subpopulations were formed based on the availability of: (A) liver tissue (55 fetuses [21,22]) and (B) plasma measurements (60 fetuses). Of the 60 fetuses in (B), 18 fetuses overlapped with the 55 livers in (A). Within each subpopulation, the studied fetuses were matched for gestational age, fetal sex, and maternal smoking/fetal plasma cotinine (Table 1). The aim of this matching was to ensure that each of the four main analytical groupings (male, non-smoke-exposed; male, smoke-exposed; female, non-smoke-exposed; female, smoke-exposed) contained similar numbers of fetuses representing similar gestational ages, enabling two-way ANOVA to be used (see Statistical analysis section below). We were unable to collect data on maternal diet under the study ethics but the women were matched for age and body mass index across the four main analytical groupings (Table 1).

**Table 1:** **Morphological data for the mothers and fetuses (mean ± SEM) at census date and then for those allocated to the liver and plasma cysteine and homocysteine analyses populations**

**Population**	**Characteristic**	**Male fetuses**		**Female fetuses**	
		**Control**	**Smoke-exposed**	**Control**	**Smoke-exposed**
Whole population at census date	*N*	57	66	49	56
Maternal indices	Age (years)	24 ± 1	25 ± 1	24 ± 1	24 ± 1
	Body mass index (BMI) (kg/m^2^)	25 ± 1	25 ± 1	24 ± 1	25 ± 1
	Cigarettes/day	0	11 ± 1	0	12 ± 1
Fetal indices	Age (weeks)	14.5 ± 0.3	15.1 ± 0.3	14.9 ± 0.3	14.9 ± 0.3
	Weight (g)	73 ± 10^a^	95 ± 9^b^	87 ± 11	92 ± 12
	Crown-rump length (CRL, mm)	94 ± 4^a^	106 ± 4^b^	101 ± 4	102 ± 4
	Ponderal index (weight g/[CRL cm^3^])	0.067 ± 0.002	0.067 ± 0.002	0.069 ± 0.003	0.067 ± 0.002
	Plasma cotinine (ng/mL)	3 ± 1^a^	40 ± 2^b^	4 ± 1^a^	43 ± 2^b^
Sub-population: methylation and metal	*N*	14	16	14	11
Maternal indices	Age (years)	21 ± 1	26 ± 2	25 ± 1	26 ± 2
	BMI (kg/m^2^)	25 ± 2	26 ± 1	24 ± 1	25 ± 2
	Cigarettes/day	0	16 ± 2	0	16 ± 2
Fetal indices	Age (weeks)	14.0 ± 0.6	14.3 ± 0.4	15.3 ± 0.7	15.8 ± 0.9
	Weight (g)	62 ± 18	74 ± 14	100 ± 20	151 ± 34
	CRL (mm)	93 ± 8	97 ± 6	103 ± 8	121 ± 9
	Ponderal index (weight g/[CRL cm^3^])	0.063 ± 0.004	0.070 ± 0.003	0.080 ± 0.007	0.071 ± 0.003
	Plasma cotinine (ng/mL)	3 ± 1^a^	44 ± 3^b^	3 ± 1^a^	48 ± 3^b^
Sub-population: plasma cysteine and homocysteine	*N*	14	17	13	16
Maternal indices	Age (years)	25 ± 2	24 ± 1	24 ± 2	24 ± 1
	BMI (kg/m^2^)	26 ± 2	25 ± 2	24 ± 1	24 ± 1
	Cigarettes/day	0	13 ± 1	0	11 ± 1
Fetal indices	Age (weeks)	15.2 ± 0.7	14.9 ± 0.6	15.6 ± 0.7	15.4 ± 0.6
	Weight (g)	102 ± 24	89 ± 18	105 ± 21	101 ± 19
	CRL (mm)	108 ± 8	105 ± 7	109 ± 8	121 ± 9
	Ponderal index (weight g/[CRL cm^3^])	0.074 ± 0.005	0.064 ± 0.002	0.065 ± 0.003	0.065 ± 0.003
	Plasma cotinine (ng/mL)	5 ± 1^a^	44 ± 3^b^	4 ± 3^a^	41 ± 3^b^

^a,b^Values in the same row that do not share a superscript letter are significantly different (*P* <0.05) due to maternal cigarette smoking. Absence of superscript letters indicates no significant differences.

### Hepatic metal content analysis and cobalt speciation

Livers were homogenized using a TissueLyser (Qiagen Ltd., Crawley, UK) and were freeze-dried and stored at −80°C [21]. For total element determination, 100 mg of liver was mixed with 1 mL concentrated nitric acid and pre-digested overnight. Hydrogen peroxide (2 mL) was added and the samples were digested using a MARS5 microwave oven. The digest was diluted to 10 mL with water (18 MΩ cm, MilliQ). Elemental standards for calibration were prepared by appropriate dilutions of multi-element standard Merck XXI (Merck, UK) and single element solutions of Mo (CPI, single element solutions) with 5% nitric acid. The samples and standards were measured using an Agilent 7500c inductively coupled plasma mass spectrometry (ICP-MS) with on-line addition of Rh-solution (20 μg/L) as an internal standard. The ICP-MS was used with H_2_ as reaction gas to remove molecular interferences of ArCl on As (m/z 75) and ArAr on Se (m/z 78). Certified standard reference materials (RM8415, TORT-2, DORM-3, BCR-185R, NIST 1,577b) were used to check reproducibility and accuracy, with both shown to be better than 5%. The hepatic essential element content was expressed as ng/g dry matter (dm). Cobalamin in its vitamin B12 form was extracted from homogenized liver (30 to 100 mg) using 2.5 mg papain and 10 μL 1% NaCN dissolved in 600 μL acetate buffer (pH 4.0). The mixture was heated to 57°C for 3 h and subsequently boiled in a water-bath for 10 min and centrifuged. Different cobalt species were separated using an Agilent Eclipse XDB-C8 (150 × 4.6 mm) column with an eluent containing 0.1% (v/v) formic acid in a gradient of 10% to 70% MeOH in 20 min. The column was held at 30°C in a column oven, the injection volume was 100 μL, and the flow rate was 1 mL/min. Cobalt detection was performed through an Element 2 ICP-MS (Thermo, Bremen). The internal standard (Rh in 1% nitric acid) was added post-column via a T-piece before the nebulizer. The column effluent was split after the column with three quarters going into the UV/electrospray ionization (ESI)-MS and one quarter into the ICP-MS using a short length of peek tubing. ESI-MS (Orbitrap Discovery, Thermo) was used for some samples in positive ES-FT-mode (resolution 30,000) from 100 to 2,000 m/z using MS/MS in FT-mode (resolution 7,500) to aid identification of the eluting vitamin B12. Quantification was performed after calibration with a cobalt standard solution and corrected for the different elution conditions for the gradient [25]. The limits of detection for every cobalamin species was 0.9 μg Co/kg dm or 1.5 nmol/kg (calculated from 3 × SD) while reproducibility was better than ±15%.

### Plasma cysteine and homocysteine determination

Fetal plasma homocysteine and cysteine were analyzed using an Agilent 1100 HPLC system (Agilent Technologies, Stockport, UK) according to previously published in-house protocols [26] based on the original method of Pfeiffer et al. [27]. Peak integration was performed using Agilent ChemStation software (Agilent Technologies).

### Real-time quantitative PCR

Total RNA was extracted from frozen fetal liver samples (10 to 20 mg) using TRIzol (Life Technologies, Paisley, UK). Reverse transcription, primer design, and real-time PCR were as previously described [28-30] and the primers used are shown in Additional file 1: Table S1. Data were normalized against a validated combination of housekeeping genes (B2M, PMM1, TBP) as described previously [21].

### Analysis of DNA methylation

**Figure 1:**
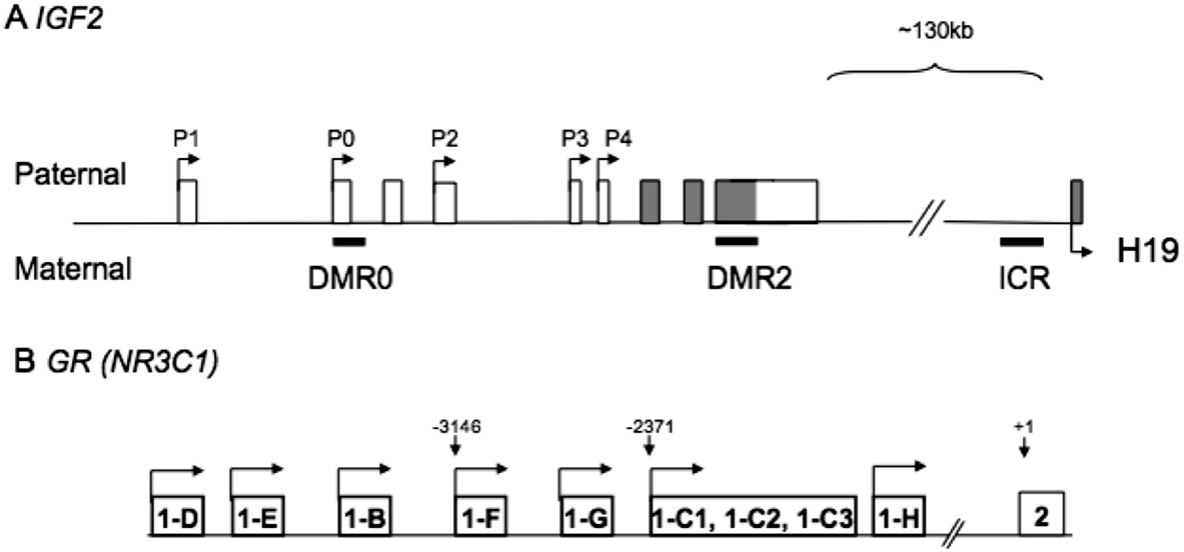
**Schematic diagrams showing of regions in which methylation was assessed including IGF2 and exons 1(F) and 1(C) of the** ***GR*** **promoter. (A)** The *IGF2* gene comprises multiple transcripts originating from promoters (P0-P4) which splice into common protein-coding exons (shaded in grey). The H19 ICR is situated distal to IGF2. Regions of differential methylation are shown underlined. **(B)** The *GR* gene also comprises multiple alternate untranslated first exons as indicated (B-H) [33]. Percentage methylation was assessed at CpGs within exons 1F, 1-C1 and 1-C3.

DNA was extracted from fetal liver using a Qiagen AllPrep DNA/RNA/Protein mini kit (Qiagen) following tissue homogenization using a Qiagen TissueLyser (Qiagen) and following the manufacturer’s instructions [31]. Briefly, 1 μg of DNA was subjected to bisulfite conversion using the Epitect Bisulfite Kit (Qiagen). Pyrosequencing was performed to analyze DNA methylation across a number of regions (Figure 1) as previously described for regions known to be important in controlling the expression of insulin-like growth factor 2 (IGF2): the IGF2 differentially methylated regions (DMRs) and the H19 imprinting control region (ICR) [32] and for exons 1(C) and 1(F) of the *NR3C1* glucocorticoid receptor (GR) promoter [31]. All primers were purchased from Eurogentec (Southampton, UK). Pyrosequencing was carried out using SQA reagents (Qiagen) on the PSQ™ HS-96A. Data were analyzed using Pyro Q-CpG Software (Qiagen). Background non-conversion levels were determined by inclusion of non-cytosine-guanine dinucleotide (CpG) cytosine controls in all assays and were between 1 and 3%.

### Statistical analysis

Statistical analyses of data were performed using JMP 9.0.2 software (Thomas Learning, London, UK) [21,22]. Normality of data distribution was tested with the Shapiro-Wilk test and non-normally distributed data were log-transformed and re-checked for normality prior to analysis by ANOVA and Tukey-Kramer honestly significant difference and T-tests. Where data were not normalized, or the variances remained unequal, non-parametric tests were performed (Wilcoxon Test). Care was taken to ensure that no comparison involved groups with any difference between smokers and non-smokers in terms of stage of gestation, avoiding bias. Because most of the parameters investigated showed developmental changes in expression across the second trimester, two-way ANOVA was used to test the combined effects of gestational age (weeks) and maternal smoking (yes/no and confirmed by fetal plasma cotinine assay) on morphological and biochemical data and on transcript levels.

## Results

### 1-carbon metabolism

#### Effects of fetal sex

**Figure 2:**
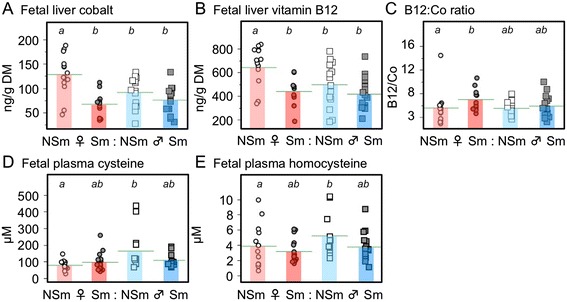
**Concentrations of cobalt (Co) and vitamin B12 (B12) in the human fetal liver. (A)** and **(B)** show the effects of sex and maternal smoking on Co and B12, respectively. The ratio between B12 and Co **(C)** was lower in females and increased to male levels by smoke-exposure in females. Circulating fetal cysteine **(D)** and homocysteine **(E)** concentrations were significantly higher in male fetuses and this was lost when the mothers smoked. Data from individual fetuses (**A–** **D**: 11–16 fetuses per group; **E–** **F**: 13–17 fetuses per group) are shown, and the horizontal bar represents the mean of the group. Significant differences between groups are indicated by letters in the boxes above each graph. Within each element or compound, groups that do not share a letter are significantly (*P* <0.05) different. NSm, represents control fetuses from non-smoking mothers; Sm, represents smoke-exposed fetuses from mothers who smoked during pregnancy. Fetal sex is denoted by the appropriate symbol.

Vitamin B12 is an essential component of the 1-carbon cycle; it is required for the transfer of the C1 unit from methyltetrahydrofolate to methionine by methionine synthase (MTR; EC 2.1.1.13), and is a rate-limiting co-factor for methionine synthase reductase (MTRR; EC 1.16.1.8). Levels of vitamin B12 and cobalt (the central metal ion in vitamin B12) were significantly lower in liver from non-exposed male fetuses than in non-exposed female fetuses (Figure 2A, B). Vitamin B12 acts as a co-factor for the conversion of homocysteine to methionine and homocysteine is converted to cysteine through the actions of cystathionine-β-synthase (EC 4.2.1.22) and cystathionine gamma-lyase (CTH; EC 4.4.1.1). We therefore also measured fetal plasma homocysteine and cysteine. Plasma homocysteine and cysteine levels were higher in non-exposed males compared to females (Figure 2D, E).

### Effects of smoke exposure

In females, both vitamin B12 and cobalt concentrations correlated significantly and inversely with fetal cotinine (r = −0.593, *P* = 0.002 and r = −0.510, *P* = 0.009, respectively) and levels of both were significantly reduced by maternal cigarette smoking to resemble the levels seen in males (Figure 2A, B and Additional file 2: Table S2). In contrast, maternal smoking was associated with a significant increase in the vitamin B12/cobalt ratio across the second trimester in males (Additional file 3: Figure S1). This reflects a decrease in the proportion of cobalt that is not bound as cobalamin and demonstrates that maternal smoking reduces non-cobalamin cobalt uptake. We also measured fetal plasma homocysteine and cysteine (to which homocysteine is converted through the actions of cystathionine-β-synthase and CTH). The sex differences in plasma homocysteine and cysteine were abolished by exposure to maternal smoking (Figure 2D, E).

### Expression of 1-carbon cycle genes and DNA methyltransferases

**Figure 3:**
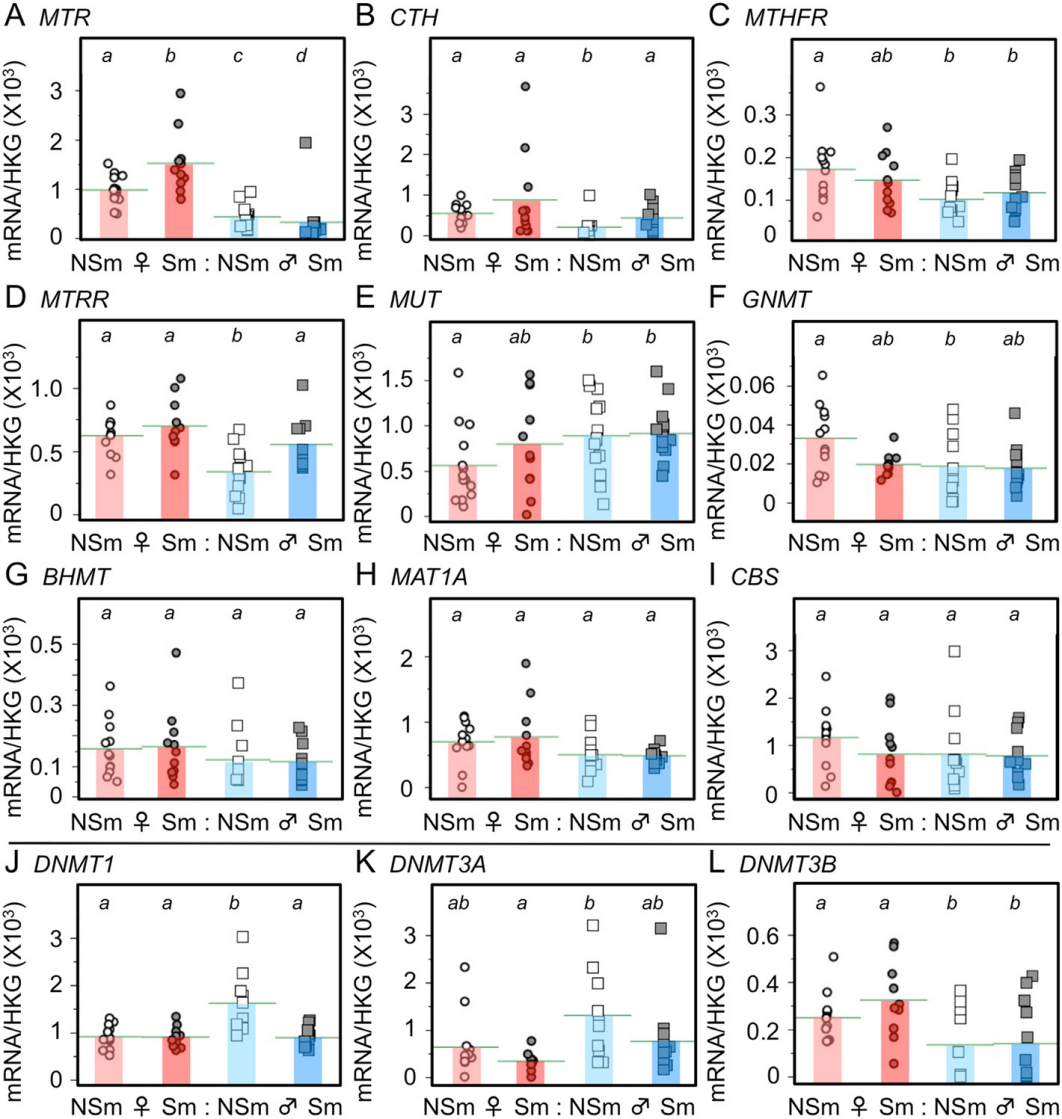
**Expression of selected transcripts encoding members of the 1-Carbon cycle (A-I) and DNA methylation enzymes, (J) DNMT1, (K) DNMT3A, (L) DNMT3B, in the human fetal liver and effects of sex and maternal smoking.** Real-time PCR was used to measure specific transcript levels as described in the text. Data from individual fetuses (11–16 fetuses per group) are shown, and the horizontal bar represents the mean of the group. Significant differences between groups are indicated by letters in the boxes above each graph. Within each transcript, groups that do not share a letter are significantly (*P* <0.05) different. NSm, represents control fetuses from non-smoking mothers; Sm, represents smoke-exposed fetuses from mothers who smoked during pregnancy. Fetal sex is denoted by the appropriate symbol.

Alterations in the expression of key genes in the 1-carbon cycle (Additional file 4: Figure S2) might alter the availability of methyl groups and impact on DNA methylation. We therefore measured the expression of mRNA transcripts encoding enzymes in the 1-carbon cycle in fetal liver (Figure 3A–I).

### Effects of fetal sex

Sex differences in transcript levels were apparent, with lower expression of *MTR*, *MTRR*, methylene-THF-reductase (*MTHFR*), glycine N-methyltransferase (*GNMT*), and *CTH* in non-exposed males compared to females. In addition, expression of the DNA methyltransferase *DNMT1* was higher and the expression of *DNMT3B* was lower in males compared to females (Figure 3J–L).

### Effects of smoke exposure

Exposure to maternal smoking was associated with sex-specific changes in mRNA transcript levels: in males, smoke exposure resulted in an increase in the expression of *CTH* and *MTRR* to female levels but also resulted in a decrease in the expression of *MTR.* In females, smoke exposure resulted in an increase in the expression of *MTR* and a decrease in the expression of *GNMT* to male levels. The expression of *DNMT1* in males was reduced to female levels by exposure to maternal smoking but there were no effects of smoking on the expression of *DNMT3A or DNMT3B* (Figure 3J–L).

### Expression of IGF2 and NR3C1 (GR)

#### Effects of fetal sex

**Figure 4:**
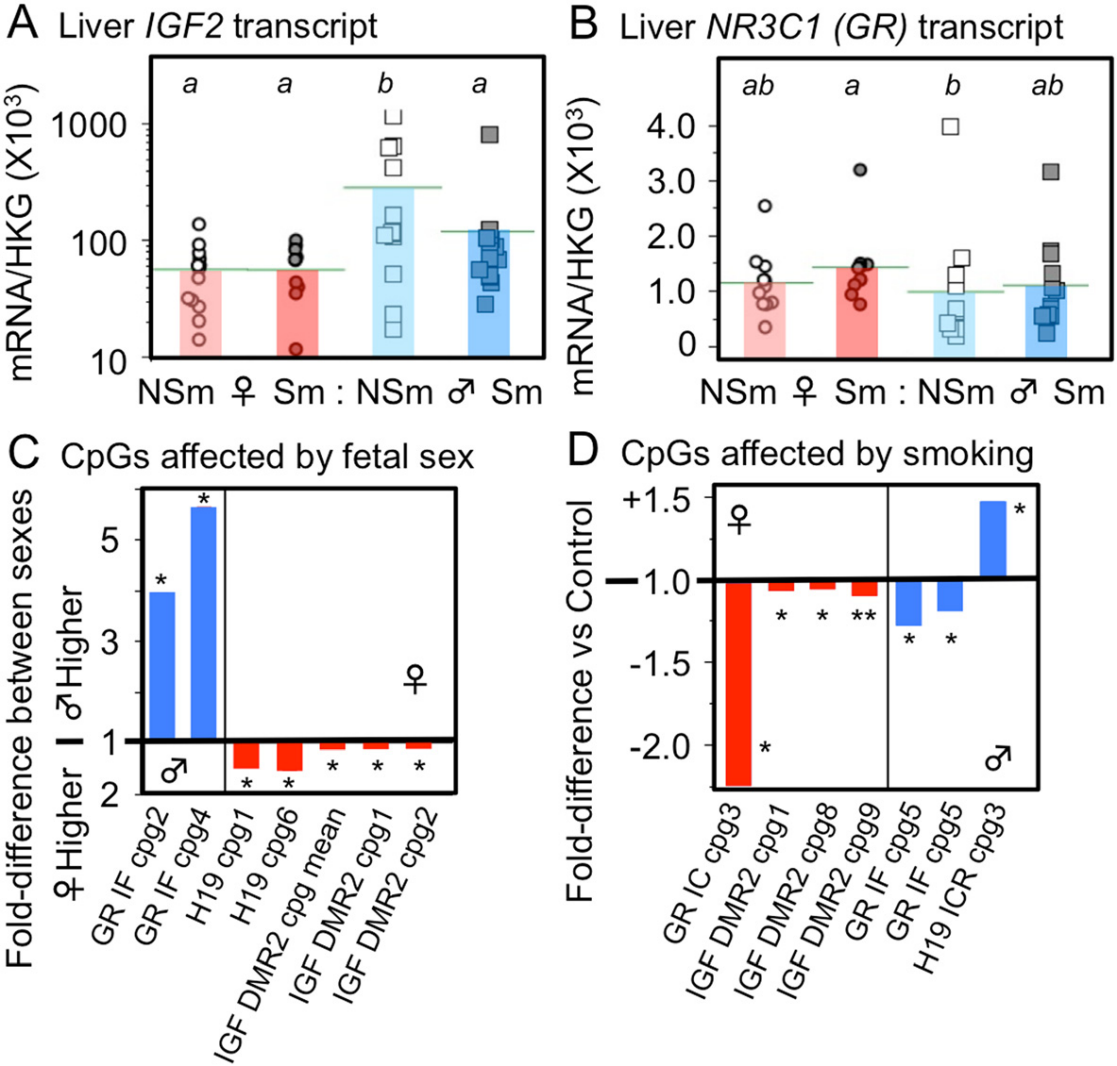
**Effects of fetal sex and maternal smoking on expression of transcripts encoding** ***IGF2*** **(A) and** ***GR*** **(B), and on DNA methylation (C and D) in the human fetal liver.** Real-time PCR was used to measure specific transcript levels and pyrosequencing was used to analyze DNA methylation as described in the text. Data from individual fetuses (11–16 fetuses per group) are shown, and the horizontal bar represents the mean of the group. In A and B, significant differences between groups are indicated by letters in the boxes above each graph. Within each transcript, groups that do not share a letter are significantly (*P* <0.05) different. NSm, represents control fetuses from non-smoking mothers; Sm, represents smoke-exposed fetuses from mothers who smoked during pregnancy. Fetal sex is denoted by the appropriate symbol.

The expression of the *IGF2* transcript was higher in males compared to females (Figure 4A) whereas expression of the glucocorticoid receptor (*GR/NR3C1*) was lower in males (*P* = 0.022, Figure 4B) compared to females.

### Effects of smoke exposure

The expression of *IGF2* transcript was decreased by smoke exposure in males but there was no effect in females (Figure 4A). There was no effect of smoking on the expression of *GR* (Figure 4B).

### DNA methylation of IGF2 and NR3C1 (GR)

Pyrosequencing was used to quantify DNA methylation at the DMRs known to be important in the control of *IGF2* expression: DMR0, DMR2, and the *H19* ICR, which lies upstream of the *H19* gene. There was a significant (*P* <0.05) increase in DNA methylation at *IGF2* DMR0 with gestational age (Additional file 5: Figure S3A). Levels of DNA methylation at exons 1C and 1F of the *GR/NR3C1* promoter were very low as previously published [31], consistent with the status of the *GR* promoter as a dense CpG island. Methylation at exon 1C increased with gestational age (Additional file 5: Figure S3B).

### Effects of fetal sex

Sex differences in DNA methylation were apparent at the H19 ICR and IGF2 DMR2, where methylation was lower in males than females. Conversely, at the *GR* promoter there was higher DNA methylation within exon 1F (with a similar trend for exon IC, *P* = 0.056) in males, consistent with the lower transcript levels of this gene (Figure 4C).

### Effects of smoke exposure

In males, smoke exposure significantly increased DNA methylation at one CpG within the *H19* ICR (Figure 4D), whereas in females, smoke exposure was associated with decreased methylation at several CpGs within *IGF2* DMR2. Additionally, at the *GR* promoter, there were small changes in DNA methylation associated with maternal smoking at specific CpGs within exon 1F in males and at one CpG in exon 1C in females (Figure 4D).

## Discussion

Vitamin B12 is an essential component of the 1-carbon cycle and data from both human and animal studies strongly support the concept that maternal and/or fetal vitamin B12 availability has important influences on health outcomes, including fetal growth, neurodevelopment, and longer term cardiometabolic disease risk [34-36]. Herein, we show, for the first time, that maternal smoking, which is known to reduce the concentrations of vitamin B12 in pregnant women and their infants [17,18], is associated with widespread effects on 1-carbon metabolism, including alterations in levels of enzyme transcripts and co-factors. Importantly, we also show that these effects are present during early development and in a key target tissue, the fetal liver.

The precise role of *in utero* methyl donor availability and/or effects on the 1-carbon pathway in the programming of later disease risk remains unknown; however, one important mechanism may be through changes in DNA methylation [11]. In animal models, dietary availability of methyl donors during pregnancy has a profound influence on both phenotype and DNA methylation in offspring [12,37]. In humans, maternal vitamin B12 concentrations correlate inversely with global DNA methylation in umbilical cord blood [15], and an inverse correlation between cord plasma homocysteine concentrations and genome-wide DNA methylation at repetitive sequences has been reported [38]. Additionally, genome-wide and candidate gene studies have identified alterations in DNA methylation in association with maternal smoking in accessible tissues at birth, including cord blood, buccal cells, and placenta [8-10]. Our findings that maternal smoking is associated with effects on DNA methylation strengthen and extend previous studies by showing that such changes are detectable in the fetal liver, a major target organ for developmental programming effects [39,40], and that they are present during early development.

The imprinted gene *IGF2* has a major role in mediating fetal growth [41] and altered DNA methylation at the *IGF2* DMRs is known to be associated with human syndromic growth disorders, including Silver Russell and Beckwith Wiedemann syndromes [42]. Recent data suggest that more subtle alterations in DNA methylation at *IGF2* are associated with patterns of fetal growth within the normal range [43-46] and some studies report that infants born small for gestational age have reduced DNA methylation at *IGF2* in cord blood [43]. Thus, reduced *IGF2* methylation in fetal liver during early-mid gestation could be one mechanism for the reduction in birthweight seen with prenatal smoke exposure. DNA methylation at *IGF2* appears to be particularly sensitive to the prenatal environment [9,47,48] and our data support the concept that methylation at *IGF2* may be a useful marker of *in utero* exposures [9]. DNA methylation at *GR* may also be influenced by diverse environmental cues in early life and animal and human studies suggest that downstream effects on the expression of *GR* may play a role in mediating the associations between the early life environment and subsequent cardiometabolic and neuropsychiatric disorders [31,39,49,50]. The methylation changes we report at both *IGF2* and *GR* in association with smoke exposure are relatively small; however, they resemble those described in previous studies, including studies of prenatal smoke exposure [9,47,51]. Additionally, the observed alterations in DNA methylation were region- and CpG site-specific, consistent with reports in both animal models and human studies [31,52]. In agreement with previously published studies in humans, DNA methylation levels did not necessarily predict changes in transcript levels, notably at *IGF2*, suggesting that there are alternative and/or additional mechanisms driving changes in transcript expression [53]. Rather, such changes in DNA methylation may result in genes being ‘poised’ for activation in response to future events [53,54].

**Figure 5:**
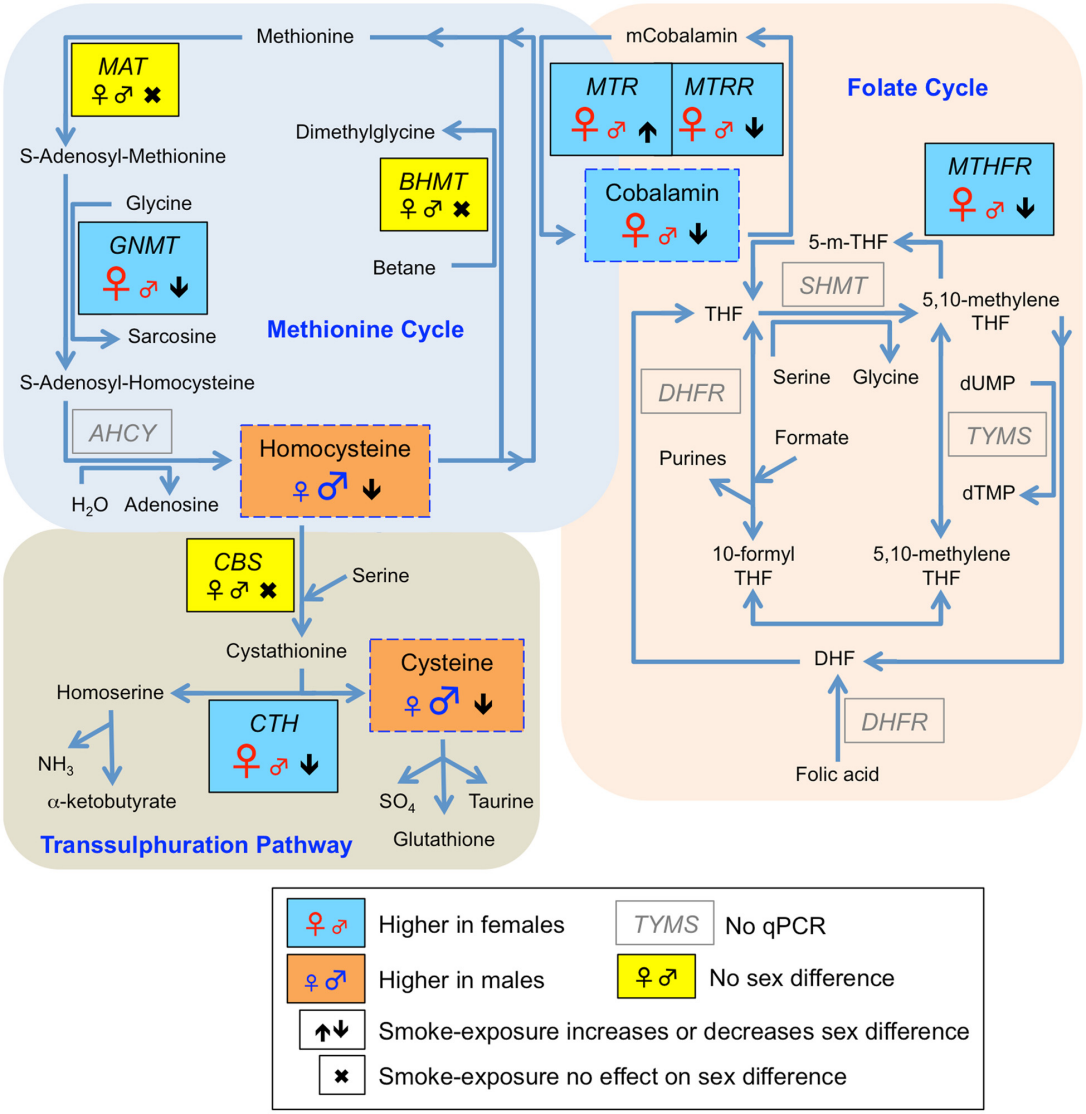
**Summary of effects of fetal sex and maternal smoking on the human fetal second trimester liver 1-carbon metabolism pathway.** Redrawn and modified from [10]. In 7 out of 8 genes or compounds showing sex differences, maternal smoking reduced the magnitude of the difference. Together with sex differences and sex-specific effects of maternal smoking on DNA methylation this demonstrates the sensitivity of the fetal liver to maternal smoking.

Sex differences were evident in DNA methylation, which was higher at *IGF2* and lower at *GR* in females compared with males and the most marked changes in DNA methylation in association with maternal smoking occurred at these loci. Sexually dimorphic changes in DNA methylation have been reported as a consequence of early life nutritional challenges and exposure to maternal smoking [9,51,55,56]. The proposed mechanisms to explain these observations include differences in the timing and speed of development, the influence of sex steroids, especially androgens, and/or sex chromosome complement [22,57]. Our results suggest that sex differences in the concentrations of vitamin B12 and homocysteine and in the expression of genes important in 1-carbon metabolism and DNA methylation could also be important in determining the sex-specific effects of exposure to an adverse environment in early life. Consistent with our previous studies [21,22], the smoke-induced changes in cobalt, vitamin B12, and homocysteine, and in the expression of enzymes involved in both the 1-carbon cycle and DNA methylation, resulted in profiles which resembled those seen in the opposite sex (summarized in Figure 5). These clearly suggest that smoke exposure results in masculinization of the female liver and feminization of the male liver (Figure 5).

Temporal changes in gene expression and DNA methylation indicate that there may be specific windows of susceptibility during development and, importantly, that this is likely to differ between the sexes. Allelic methylation patterns at the *IGF2* DMRs arise early in embryogenesis and change progressively during development [58] and here we show that this is also the case for *GR* between 12 and 20 weeks of gestation. Thus, since a worryingly high proportion of women continue to smoke throughout pregnancy, the observed effects on DNA methylation may change or become amplified with ongoing exposure. Additionally, the temporal changes in DNA methylation profiles across gestation could be one explanation for the differences between studies with respect to the association of DNA methylation at *IGF2* and fetal growth [9,59]. Whilst the data shown here represent a single, albeit unique, 10-week snap-shot of the effects of maternal smoking, they are consistent with previous studies showing that maternal smoking is associated with altered DNA methylation in blood, buccal cells, and placenta in exposed offspring at birth [8]. Importantly, these changes may be persistent and/or evolve postnatally since DNA methylation patterns in peripheral blood and buccal cells in young children and in peripheral blood in adolescence and young adulthood also associate with *in utero* exposure to maternal smoking [7,10,60,61]. Furthermore, some studies report that methylation differences only become apparent some considerable time after *in utero* environmental exposures [51].

## Conclusions

Our novel data greatly extend previous studies linking *in utero* exposures with altered DNA methylation by showing, for the first time, that such changes are present comparatively early in fetal life and, importantly, that these occur in a key metabolic target tissue, the liver. These changes are likely to have a direct effect on fetal development and, if persistent, may have long-term effects on health. Additionally these data propose an important new mechanism by which these changes might be induced, through alterations in methyl donor availability and changes in the 1-carbon cycle.

## Additional files

Additional file 1:
**Primers used for qPCR.**
Additional file 2:
**Fetal human hepatic essential element content.**
Additional file 3:
**Maternal smoking is associated with a significant increase in the ration between hepatic Co and vitamin B12 in males fetuses only across the second trimester.** Males are shown by squares, females by circles, controls are open, smoke-exposed are shaded. “NSm” represents control fetuses from non-smoking mothers; “Sm” represents smoke-exposed fetuses from mothers who smoked during pregnancy. Fetal sex is denoted by the appropriate symbol. Additional file 4:
**Summary of the 1-carbon metabolism cycle.** Simplified and modified diagram based on Steegers-Theunissen RPM, Twight J, Pestinger V and Sinclair KD. The periconceptional period, reproduction and long-term health of offspring: the importance of one-carbon metabolism. *Human Reproduction Update* 19,640-655 (2013). Additional file 5:
**Fetal liver methylation levels of specific or mean CpGs for** ***IGF2*** **(A) and** ***GR*** **(B) icrease significantly across the second trimester.** Males are shown by squares, females by circles, controls are open, smoke-exposed are shaded.

## Abbreviations

CpG: Cytosine-guanine dinucleotide
CTH: Cystathionine gamma-lyase
DMR: Differentially methylated region
DNMT: DNA methyltransferase
ESI: Electrospray ionization
GNMT: Glycine N-methyltransferase
GR: Glucocorticoid receptor (*NR3C1*)
ICP-MS: Inductively coupled plasma mass spectrometry
ICR: Imprinting control region
IGF2: Insulin-like growth factor 2
MTHFR: Methylene-THF-reductase
MTR: methionine synthase
MTRR: Methionine synthase reductase
