# Supplementary material for: In utero exposure to cigarette chemicals induces sex-specific disruption of one-carbon metabolism and DNA methylation in the human fetal liver
Source: BMC Med. 2015 Jan 29;13:18. doi: 10.1186/s12916-014-0251-x (PMC4310040; doi:10.1186/s12916-014-0251-x)
Supplement: Additional file 1: — Primers used for qPCR. [file 12916_2014_251_MOESM1_ESM.docx]

**Table S1** Primers used for qPCR.

| **Gene name** | **Gene** | **Forward** | **Reverse** |
| --- | --- | --- | --- |
| Methylenetetrahydrofolate reductase (NAD(P)H) | *MTHFR* | Gagggaggcttcaactacgcagtg | tgaaatcggctcccgcagac |
| 5-methyltetrahydrofolate-homocysteine methyltransferase reductase | *MTRR* | Aacagaggttctgcggaagggagt | tctggtaagtggaaagaatttgttgttcg |
| Glycine N-methyltransferase | *GNMT* | ccagcgggtgctcgacgtag | cccacttgtcgaaggcggg |
| Cystathionase (cystathionine gamma-lyase) | *CTH* | Ttcgccacgcaggcgatc | aaggcaattcctagtgggatttccag |
| 5-methyltetrahydrofolate-homocysteine methyltransferase | *MTR* | atgctccccggcctatctttatttc | aaaaggtctcatttcagctgcaccc |
| Insulin-like growth factor 2 (somatomedin A) | *IGF2* | Ttcttggccttcgcctcgtg | gccaggtcacagctgcgga |
| Glucocorticoid receptor (Nuclear receptor subfamily 3, group C, member 1) | *GR (NR3C1)* | cctggtcgaacagttttttctaatggct | gttaagactccataatgacatcctgaagcttc |
| DNA (cytosine-5-)-methyltransferase 1 | *DNMT1* | tgtgtacctgccccctgaggc | cggccaattcggtagggctc |
| DNA (cytosine-5-)-methyltransferase 3 alpha | *DNMT3A* | Gccaggccgcattgtgtctt | tgtacgtggcctggtggaacg |
| DNA (cytosine-5-)-methyltransferase 3 beta | *DNMT3B* | Gcccgccatggtggtgtct | Cttattgaaggtggccaaattaaagtgctg |
