# Supplementary material for: In utero exposure to cigarette chemicals induces sex-specific disruption of one-carbon metabolism and DNA methylation in the human fetal liver
Source: BMC Med. 2015 Jan 29;13:18. doi: 10.1186/s12916-014-0251-x (PMC4310040; doi:10.1186/s12916-014-0251-x)
Supplement: Additional file 2: — Fetal human hepatic essential element content. [file 12916_2014_251_MOESM2_ESM.docx]

**Table S2.** Fetal human hepatic essential element content. Values are expressed as mean±s.e.m, ng/g dry liver weight. Values in the same row that do not share a superscript letter are significantly different (p<0.05) due to maternal cigarette smoking or fetal sex. Absence of superscript letters indicates no significant differences.

|  | | | | |
| --- | --- | --- | --- | --- |
| **Element/Compound** | **Male fetuses** | | **Female fetuses** | |
|  | **Control** | **Smoke-exposed** | **Control** | **Smoke-exposed** |
| *n* | *14* | *16* | *14* | *11* |
| *Significantly affected by maternal cigarette smoking* | | | | |
| Co | 91.1±8.5 ^a^ | 74.6±7.2 ^a^ | 128.1±11.3 ^b^ | 68.5±7.2 ^a^ |
| B12 | 497±51 ^a^ | 417±34 ^a^ | 643±48 ^b^ | 441±36 ^a^ |
| Mn | 12.9±0.8 ^a^ | 9.9±0.7 ^b^ | 11.9±1.1 ^ab^ | 9.8±0.9 ^ab^ |
| Li | 114±7 ^a^ | 154±33 ^ab^ | 134±5 ^bc^ | 120±4 ^a^ |
| *Not significantly affected by maternal cigarette smoking* | | | | |
| Mg | 1893±100 | 1789±66 | 1848±52 | 1887±65 |
| Al | 2.1±0.1 | 2.2±0.2 | 2.3±0.1 | 2.3±0.2 |
| Fe | 6721±707 | 6193±452 | 7125±461 | 6216±536 |
| Ni | 1100±493 | 612±59 | 678±56 | 874±261 |
| Cu | 476±31 | 444±31 | 460±32 | 435±34 |
| Zn | 2045±168 | 2111±146 | 2084±95 | 1954±125 |
| As | 63.4±18.1 | 44.1±5.6 | 37.9±6.4 | 36.6±10.4 |
| Se | 3611±184 | 3552±85 | 3890±267 | 3716±136 |
| Rb | 41.0±2.7 | 37.5±3.4 | 38.0±1.9 | 38.8±2.3 |
| Sr | 656±35 ^a^ | 680±18 ^ab^ | 733±23 ^b^ | 688±31 ^ab^ |
| Mo | 700±43 | 678±27 | 689±25 | 693±37 |
| Pb | 330±29 | 309±18 | 306±15 | 305±20 |
