# Supplementary figures and images for: In utero exposure to cigarette chemicals induces sex-specific disruption of one-carbon metabolism and DNA methylation in the human fetal liver
Source: BMC Med. 2015 Jan 29;13:18. doi: 10.1186/s12916-014-0251-x (PMC4310040; doi:10.1186/s12916-014-0251-x)

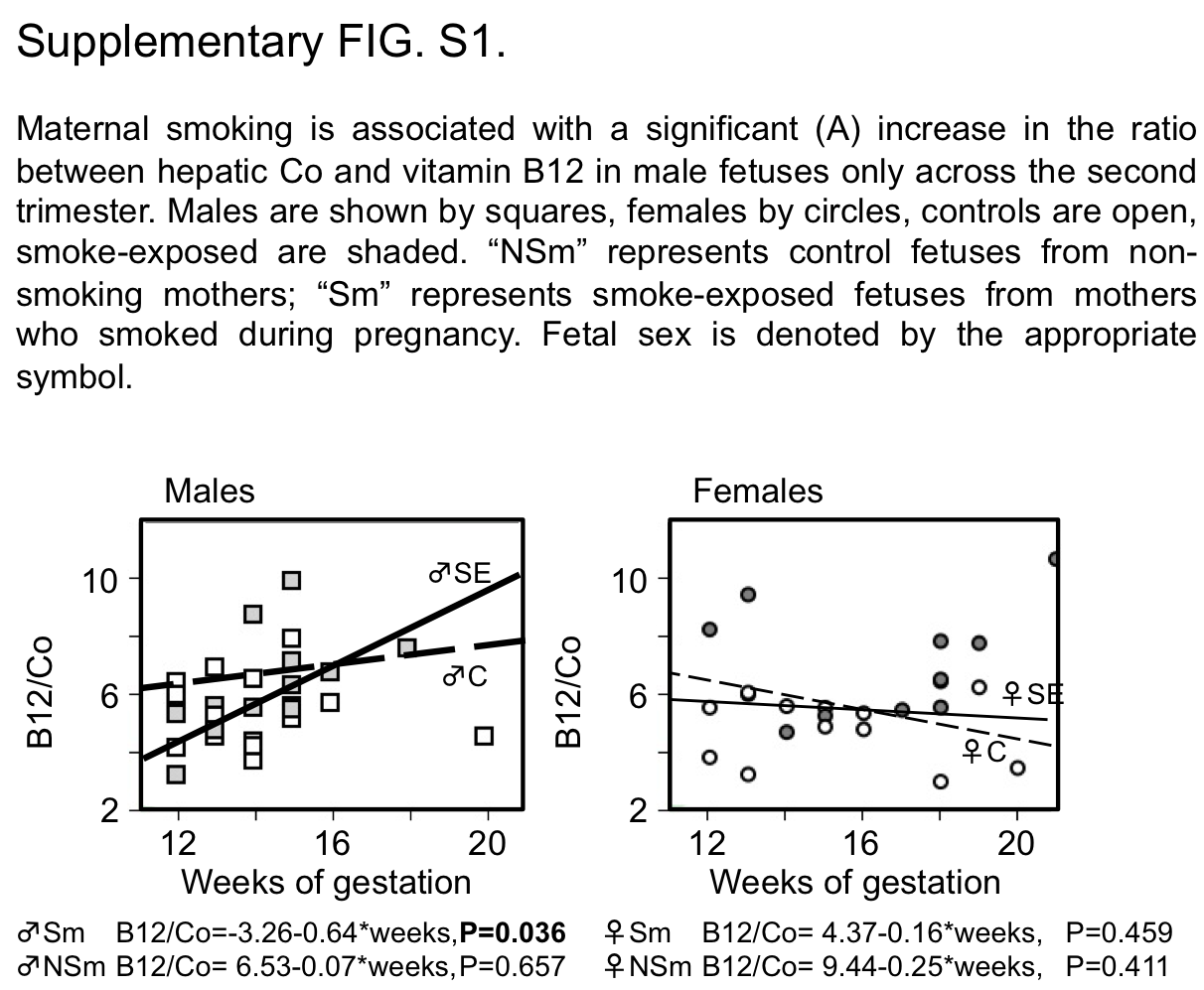

Supplement: Additional file 3: — Maternal smoking is associated with a significant increase in the ration between hepatic Co and vitamin B12 in males fetuses only across the second trimester. Males are shown by squares, females by circles, controls are open, smoke-exposed are shaded. “NSm” represents control fetuses from non-smoking mothers; “Sm” represents smoke-exposed fetuses from mothers who smoked during pregnancy. Fetal sex is denoted by the appropriate symbol. [file 12916_2014_251_MOESM3_ESM.tiff]

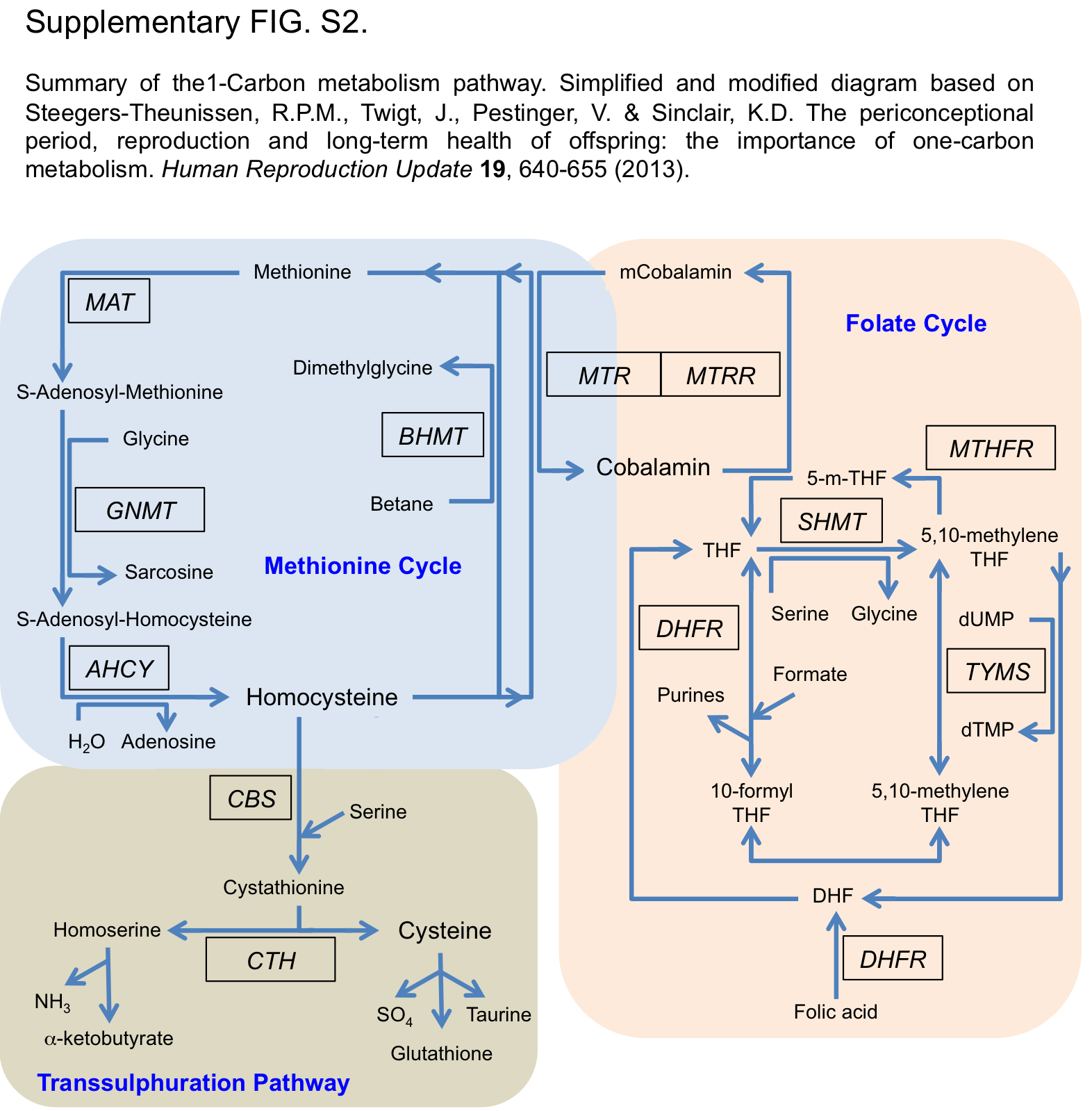

Supplement: Additional file 4: — Summary of the 1-carbon metabolism cycle. Simplified and modified diagram based on Steegers-Theunissen RPM, Twight J, Pestinger V and Sinclair KD. The periconceptional period, reproduction and long-term health of offspring: the importance of one-carbon metabolism. Human Reproduction Update 19,640-655 (2013). [file 12916_2014_251_MOESM4_ESM.tiff]

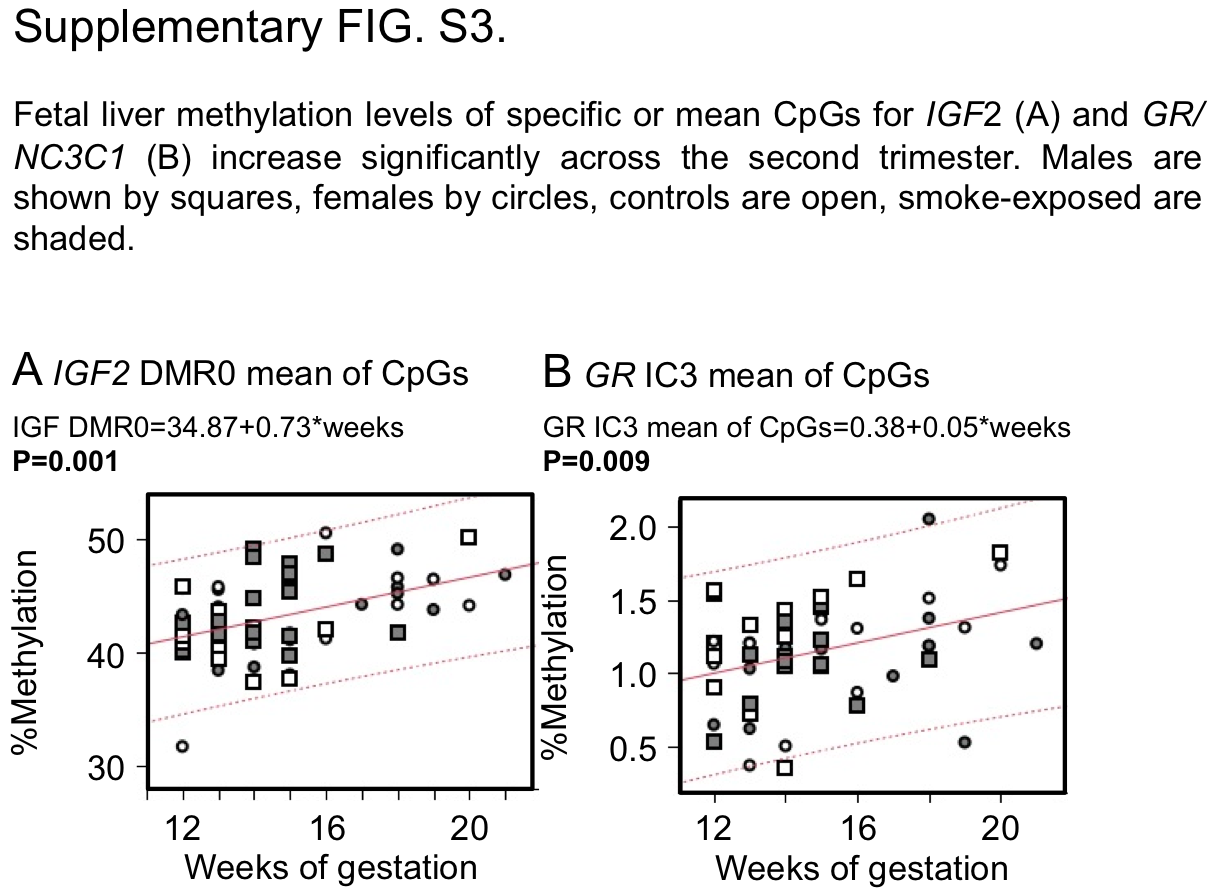

Supplement: Additional file 5: — Fetal liver methylation levels of specific or mean CpGs for IGF2 (A) and GR (B) icrease significantly across the second trimester. Males are shown by squares, females by circles, controls are open, smoke-exposed are shaded. [file 12916_2014_251_MOESM5_ESM.tiff]
